# Privacy-Preserving Predictive Modeling: Harmonization of Contextual Embeddings From Different Sources

**DOI:** 10.2196/medinform.9455

**Published:** 2018-05-16

**Authors:** Yingxiang Huang, Junghye Lee, Shuang Wang, Jimeng Sun, Hongfang Liu, Xiaoqian Jiang

**Affiliations:** ^1^ Health Sciences Department of Biomedical Informatics University of California - San Diego La Jolla, CA United States; ^2^ School of Management Engineering Ulsan National Institute of Science and Technology Ulsan Republic Of Korea; ^3^ Department of Biomedical Informatics University of California - San Diego La Jolla, CA United States; ^4^ Department of Industrial and Management Engineering Pohang University of Science and Technology Pohang Republic Of Korea; ^5^ School of Computational Science and Engineering at College of Computing Georgia Institute of Technology Atlanta, GA United States; ^6^ Department of Health Sciences Research Mayo Clinic College of Medicine Rochester, MN United States

**Keywords:** interoperability, contextual embedding, predictive models, patient data privacy

## Abstract

**Background:**

Data sharing has been a big challenge in biomedical informatics because of privacy concerns. Contextual embedding models have demonstrated a very strong representative capability to describe medical concepts (and their context), and they have shown promise as an alternative way to support deep-learning applications without the need to disclose original data. However, contextual embedding models acquired from individual hospitals cannot be directly combined because their embedding spaces are different, and naive pooling renders combined embeddings useless.

**Objective:**

The aim of this study was to present a novel approach to address these issues and to promote sharing representation without sharing data. Without sacrificing privacy, we also aimed to build a global model from representations learned from local private data and synchronize information from multiple sources.

**Methods:**

We propose a methodology that harmonizes different local contextual embeddings into a global model. We used Word2Vec to generate contextual embeddings from each source and Procrustes to fuse different vector models into one common space by using a list of corresponding pairs as anchor points. We performed prediction analysis with harmonized embeddings.

**Results:**

We used sequential medical events extracted from the Medical Information Mart for Intensive Care III database to evaluate the proposed methodology in predicting the next likely diagnosis of a new patient using either structured data or unstructured data. Under different experimental scenarios, we confirmed that the global model built from harmonized local models achieves a more accurate prediction than local models and global models built from naive pooling.

**Conclusions:**

Such aggregation of local models using our unique harmonization can serve as the proxy for a global model, combining information from a wide range of institutions and information sources. It allows information unique to a certain hospital to become available to other sites, increasing the fluidity of information flow in health care.

## Introduction

### Motivation

As large datasets from different areas ranging from genetics, microbiomes, nutrients, medicine, medical devices to the environment are being collected from large populations, it is believed that more efforts should be spent on reshaping the wealth of data and utilizing them to promote precision medicine [1]. The characterization of each person on a multidimensional level might lead to far more intricate diagnostic and prognostic groupings of populations and labeling of individuals [2]. Pertinent studies include finding relevant biomarkers, distinguishing patterns for rare diseases, discovering the combined effects of multiple genetic variants or epistasis, and researching the unique phenotype of diseases that only appears in certain demographics or ethnicities. All of them require a large sample size to avoid false positives and insignificant results [3,4].

To gather such large samples, there have been some efforts to share deidentified data such as clinical notes in compliance with the Health Insurance Portability and Accountability Act (HIPAA) [5]. However, permissions to access other’s data in a central warehouse are still cumbersome to obtain, and deidentification efforts are either costly, error prone, or ineffective [6]. Human-based deidentification efforts cost over 5000 hours and US $500,000 on the Medical Information Mart for Intensive Care-III (MIMIC-III) dataset [7] which contains only about 50,000 patient visits and 100 million words [8] and produces error recall ranging from 0.63 to 0.94 [9]. Machine-assisted deidentification shows varying results from time savings of 13.85% to 21.5% to results showing no improvement in either quality or time saved [10]. Machine learning, algorithm-based, automated deidentification can be very useful, but state-of-the-art deep learning–based deidentification models for unstructured data is still incapable of reaching the level of privacy protection set by HIPAA safe harbor, which has roughly a 0.013% reidentification rate [8,11]. In the biomedical community, there is an urgent need for developing a new method to share information learned from local sources to generalize and scale up research effort.

### Objective

Our objective to address the above challenges is to create a federated clinical analysis framework through the aggregation of local representations and models. Related studies have been published, focusing on not only simple analyses such as database queries with very specific inclusion or exclusion criteria, but also sophisticated algorithms for prediction analysis, including logistic regression [12,13], support vector machine [14,15], *k*-nearest neighborhood [16], Cox regression [17], and tensor factorization [18]. However, most studies involve restrictive assumptions originating from the requirement that data should be integrated in a matrix format, either common feature events assumption for horizontally partitioned data or common patient records assumption for vertically partitioned data. Both assumptions have limitations to reflect the situations in reality. For horizontally partitioned data, having common feature events is an unreasonable assumption as different hospitals may have different attributes because of different specialties. These attributes are often structured data such as International Classification of Diseases (ICD) code used for billing, or custom assigned code for prescriptions, lab tests, procedures, etc. Furthermore, different hospitals might have their own annotation systems for the same medical events because of the lack of a consistent and unambiguous terminology system. Similarly, for vertically partitioned data, having common patient records in different institutions is somewhat another unreasonable assumption as we might not expect all patients to be accurately linked together for the hospitals they visit. There is a need to develop a new model that is more realistic.

Recently, there has been considerable attention in the application of neural networks to represent medical concepts as multidimensional and continuous vectors [19,20]. A process called contextual embedding, commonly used in natural language processing, maps each word from a corpus of text to a hyperdimensional space where similar words in terms of meaning or distributed usage would be located nearby (eg, short cosine distance). In the realm of health care, given a corpus of patients’ history in a structured form, where medical events such as diagnoses, prescriptions, and lab tests are ordered chronologically for each patient, contextual embedding can embed each of these medical events so that similar events are closer in the final acquired space. Unlike one-hot representation, which may not be able to make distinction between related concepts such as congestive heart failure and myocardial infarction, contextual embedding produces a closer distance for these two concepts than other unrelated concepts (ie, kidney failure). Models that utilize such representation have shown higher prediction performance than previous models that do not [19,20]. Furthermore, research into identifying named entity recognition [21], abbreviation expansion [22], predicting unplanned hospital readmission [23], and predicting disease risk that incorporates long- or short-term dependencies in the electronic health record (EHR) [24] are examples of areas that have improved results with the application of word embedding as the first step [25]. As more deep-learning models dive into the realm of clinical text, instead of just using structured data to make predictions, word embeddings is becoming the paramount prerequisite for these studies.

Existing contextual embedding models are often built upon EHR data from a single institution. Each of these separate sites may contain information that other sites lack. It would be ideal if a model was built on raw data aggregated from different hospital sites to compensate for the missing or sparse information each site may have, but because of privacy concerns and the current state of interoperability in health care, such aggregation is often infeasible. To address these problems, we propose that each hospital builds its own contextual embedding model, after which no patient-level information would remain in the acquired representations (ie, embeddings). Then, hospitals can share their own local models and subsequently, the wealth of information from their hospitals without violating patient privacy. Such aggregation of local models can serve as the proxy for a global model, combining information from a wide range of institutions and information sources.

As each model is trained separately and lies in different embedding spaces, it is difficult to analyze events from different hospitals together even though some events might be semantically related or even identical. In this paper, we propose a methodology that harmonizes different contextual embeddings into a global model. Code can be found in [26].

## Methods

### Temporal Clinical Pathway

**Figure 1:**
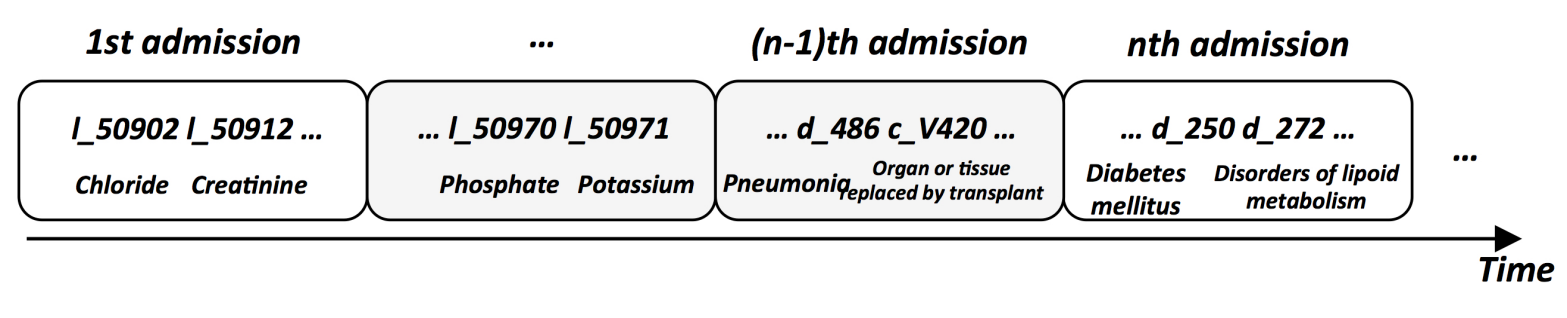
Example of a clinical pathway created from a patient’s structured data.

For this paper, we will explore structured data such as lab tests, prescriptions, symptoms, conditions, and diagnoses. We will also explore unstructured data or clinical notes. For structured data, each code was given a prefix added to differentiate them according to their type: “l_” for lab tests, “c_” for conditions, “s_” for symptoms, “d_” for diagnoses, and “p_” for prescriptions. Each of these medical events in each patient’s history was then put in chronological order to form his or her clinical pathway. An example is shown in Figure 1.

For unstructured data, Metamap [27] was used to first identify the medical concepts from free texts. Multiple words identified as a single medical concept were concatenated to form a single word. All words not mapped were omitted from the notes. No words were excluded with a cutoff score or selected for specific functionality. As long as Metamap was able to identify a word as a concept in the Metamap database, the word or words were kept. As done with the structured data, each of these medical concepts in each patient’s history were then put in chronological order to form his or her clinical pathway.

### Contextual Embedding

**Figure 2:**
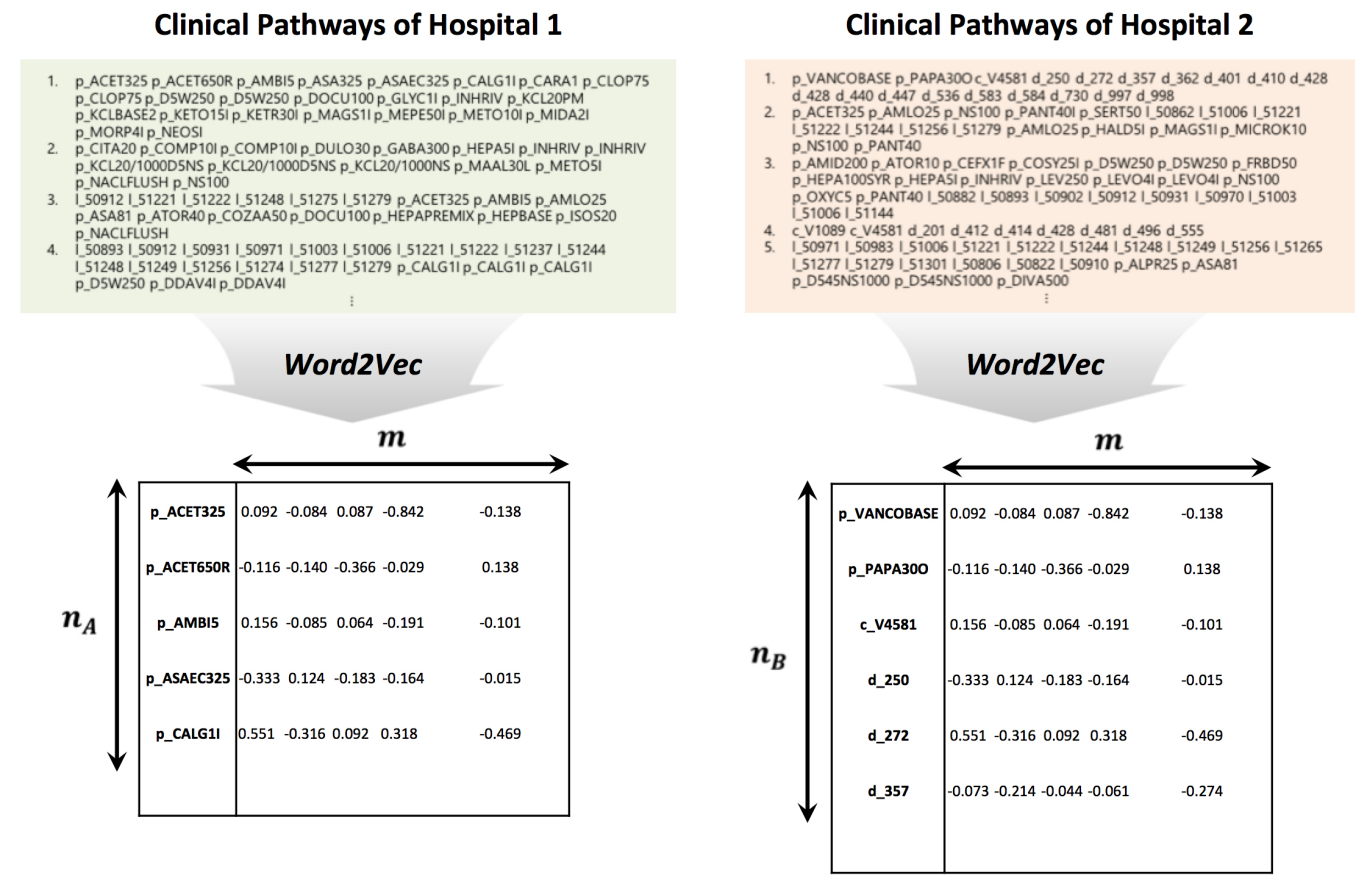
Example of two contextual embeddings created from two hospitals' structured data.

Given the clinical pathways of structured or unstructured data, we used contextual embedding to create continuous vectors for each medical event or concept, respectively. An example is shown for structured data in Figure 2 (The detailed information about the figure will be described in the following “Harmonization” section). For contextual embedding technique of this paper, we chose Word2Vec [28], which uses a neural network architecture to represent words of a large corpus as vectors. Unlike classical representation techniques such as one-hot representation, Word2Vec can effectively model words by considering the context in which the words are contained. Two architectures exist in this regard [28]—the continuous skip-gram model and the continuous bag-of-words (CBOW) model—depending on how the neural network is configured. Both architectures are essentially a three-layer network consisting of input, projection, and output layers. Providing the input as a sequence of a 1-of-*M* coding, where *M* is the vocabulary size, Word2Vec is capable of projecting them into a lower dimensional space while extracting their context. For this paper, we chose the skip-gram model for its accuracy. Experimental results for CBOW and GloVe (another embedding method) are shown in Multimedia Appendices 1-8, but skip-gram showed the best overall results. The model requires two parameters, size and window, defining the dimensionality of the final vector representation and maximum distance for contextual consideration, respectively [19,28].

There is one limitation to contextual embedding techniques such as Word2Vec and GloVe [29]. That is, because of random sampling in the training process, even repetitions on the same dataset result in embeddings of different orientations. This means that even if embeddings trained from the same dataset are pooled together naively, the medical events or concepts in one embedding would have an unreasonable relationship with events or concepts in the other embedding (eg, heart attack in one embedding would have the closest distance to elephantiasis in the other embedding).

### Harmonization

Due to the limitation of contextual embeddings, two embeddings learned from two hospital sites would lie in different hyperdimensional spaces, which makes them difficult to be used together. Therefore, there is a need to harmonize them. This is regarded as a space alignment problem [30], and it can be solved by manifold learning with or without dimensionality reduction. Manifold learning can be classified into linear and nonlinear approaches based on the assumption of data structure. Here we adopt Procrustes [31], a linear method that composes of three affine operations (transformation, rotation, and scaling) for its simplicity and generalizability. The basic idea is very similar to automatic image alignment based on scale-invariant feature transform in computer vision, although in this case, we were dealing with high dimensional attributes in this contextual embedding harmonization.

Using Procrustes to fuse different vector models into one common space requires a list of corresponding pairs [30]. These are pairs of words that are the same events but may or may not be labeled differently in different institutions. With most hospitals using standardized terminology systems such as ICD, Ninth Revision (ICD-9) and ICD-10 for billing purposes, we can reasonably identify a list of codes referring to the same events in different hospital sites to serve as our “anchor pairs” for alignment. Using these common events, we derived an orthogonal matrix that transforms one contextual embedding into the space of another.

Taking the two contextual embeddings in Figure 2 as an example, the embeddings are shown in equation 1 where *n*_A_ and *n*_B_ are the number of contextual embeddings from two hospital sites, respectively, and *m* is the dimensionality of embeddings. Taking the corresponding anchor pairs *X* and *Y*, where *X* is a subset of *A* and *Y* is a subset of *B*, we can solve for orthogonal matrix *Q* and scalar *k* from the corresponding anchor pairs in equation 1. Applying *Q* and the scalar *k*, we can solve for *A*^f^and *B*^f^, which are the harmonized vector representation of *A* and *B.*


*A∈*
*R*
^n^_A_^x m^

*B∈*
*R*
^n^_B_^x m^

*(1) Min*_Q, k_ ||
*( X* –
*1*_n_ µ
_X_^T^
*)* –
*kQ(Y* –
*1*_n_ µ
_Y_^T^ ||
_F_)

*μ*_X_^T^and *μ*_Y_^T^represent column-wise mean vectors of *X* and *Y*; *n* is the number of corresponding anchor pairs. *Q* is solved as shown in equation 2 using singular value decomposition:

(
*X –* 1
_n_
*µ*_X_^T^)
^T^(
*Y –* 1
_n_
*µ*_Y_^T^)
*= UΣ*
*V*
^T^

*Q = UV*
^T^

(2)
*k = trace(Σ) / trace(Y
^T^ Y)*

With the orthogonal matrix and scaling factor, *Q* and *k*, we can apply them onto one of the contextual embeddings to transform one into the space of the other as shown in equation 3:


*A*
^f^ =
*A –*1
_nA_
*µ*_X_^T^

(3)
*B*
^f^ =
*kQ*(
*B –*1
_nB_
*µ*_Y_^T^)

**Figure 3:**
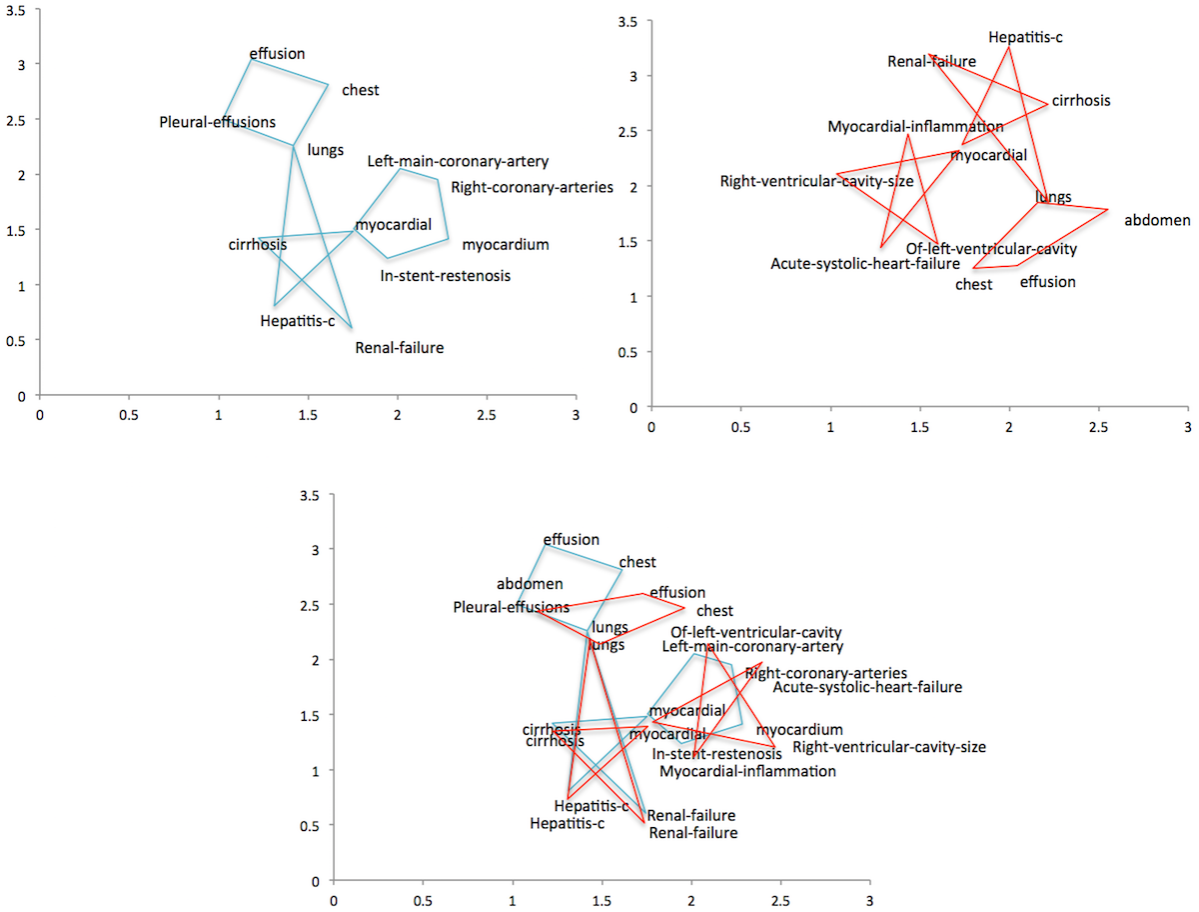
Example of Procrustes harmonization. Upper left: local embeddings of site 1. Upper right: local embeddings of site 2. Bottom: combined embeddings of two sites after harmonization.

An example is illustrated in Figure 3. We can see that “hepatitis_c,” “cirrhosis,” “lungs,” “myocardial,” and “renal_failure” in the upper left and upper right part of Figure 3 are common in both local models. Using them as anchor pairs to derive the orthogonal matrix, we harmonized the two local models into a common one shown as the bottom part of Figure 3.

### Patient Diagnosis Projection Similarity

**Figure 4:**
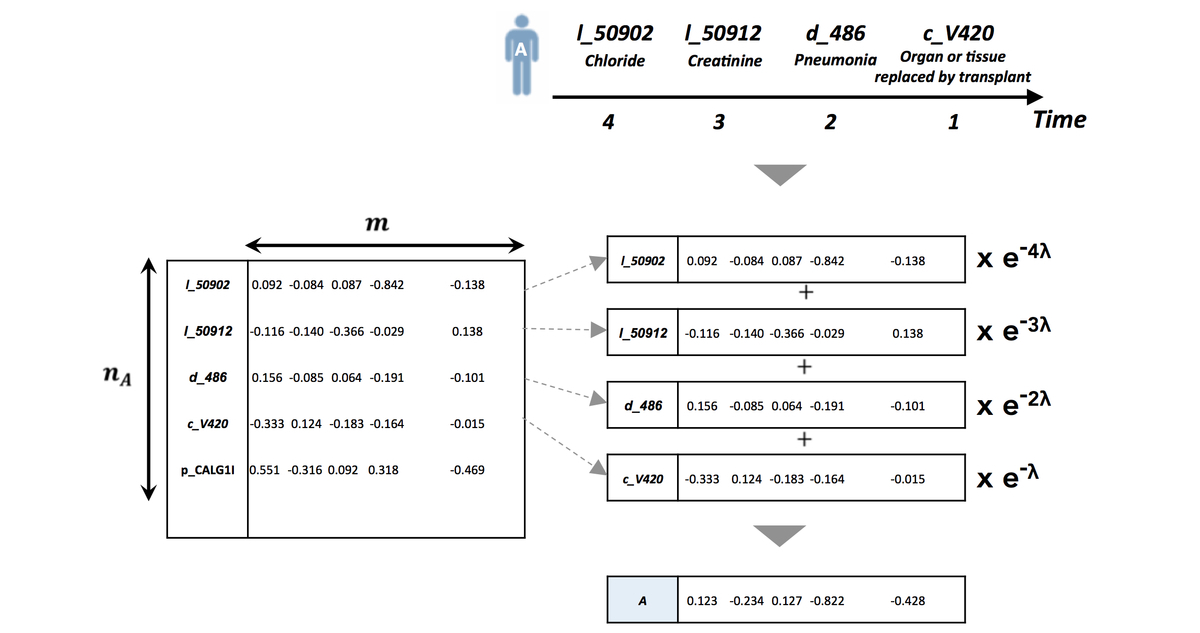
Example of creating a patient vector from event-level vector representation. The patient vector is a linear combination of the event vectors, weighted by a time decay function (e –λt with a time decay factor λ).

To predict the next likely diagnosis of a new patient for structured data experiments, we used the patient-diagnosis projection similarity (PDPS) method [19]. To calculate PDPS, we first create a patient vector. In short, we normalize the summation of each vector representation of events in the clinical pathway of a patient, with each event vector multiplied by a time decay function (ie, *e*^-λt^with a time decay factor *λ*; see Figure 4). To calculate the probability of each diagnosis occurring as the next event, we calculate the cosine similarity between the patient vector and a diagnosis vector. The equation explaining this process is shown in equation 5, where the *V*_d_ is the contextual vector representation of diagnosis *d i* n the vector space, *V*_c_ is the vector contextual representation of a medical event in the clinical pathway of a patient, *S*, and thus equation 4 is the patient vector. The number of events from the last event of the clinical pathway is *t*_c_.

(4) Σ
_c∈__S_V
_c_­
*e*¯
^λ^
^t^
^c^

(5)
*y(S, d)* =
*cosine*(
*V*_d_, Σ
_c∈__S_V
_c_­
*e*¯
^λ^
^t^
^c^)

## Results

### Data Processing

The dataset we used was from MIMIC-III, a freely accessible critical care database [7]. We had two sets of experiments. One was conducted on structure data such as codes for diagnoses, prescriptions, lab tests, symptoms, and conditions. In MIMIC-III, these structured parts of the data include everything coded that were billable. ICD-9 code was used for symptoms, diagnoses, and conditions. Custom local codes were used for lab tests and prescriptions. Another experiment was conducted on unstructured data (ie, clinical notes). For structured data, ICD-9 codes for diagnoses in MIMIC-III were generalized to level 3. For example, a patient with “diabetes with ketoacidosis, type I (juvenile type) uncontrolled” (250.13) was generalized to diabetes mellitus (250) by reducing all ICD-9 code to three digits. Because our evaluation was based on the prediction accuracy, we excluded patients who only have one admission. We also excluded rare medical events that happened in less than 50 admissions for the structured data. In the end, we kept 5639 patient records for the experiment. From these records, we constructed the temporal clinical pathway for both structured and unstructured data. Ten-fold cross validation was implemented for all experiments, which randomly splits the dataset into ten folds with equal sizes, using nine folds for training and one fold for testing.

In each replicate, to simulate two different hospital sites, we divided the training patient records into two groups of patients randomly; we call these “local” sites. Experiments done on all training patients were used as a gold standard for comparison; we call this “global.” From the training set, we created the “global” contextual embedding model using all patient records and the two “local” embedding models each using half of all patient records. The size and window parameters used to learn word embedding for structured data were 350 and 30, respectively. For unstructured data, the parameters were 350 and 100, respectively.

These two “local” embeddings were harmonized into a common model using Procrustes. As our two “local” hospital sites both came from splitting MIMIC-III [7], technically, the number of corresponding anchor pairs can account for almost the entirety of all the medical events. To create more realistic simulations, we tried different smaller fractions of all possible corresponding anchor pairs and changed the rest of the pairs artificially to be labeled differently so no events could be recognized by the other site except for the corresponding events. This was done to simulate the difference hospitals might have in their own terminology and the possibility that only a fraction of all their medical events codes are in common.

### Structured Data Results

For structured data, the harmonization of the two “local” embeddings required common events to serve as corresponding anchor pairs. There were a total of approximately 2700 total unique events between the two sites, of which there were approximately 2500 common events. We used different percentages of all possible common events as corresponding pairs for different experimental scenarios, and the rest of the pairs were artificially labeled differently, where the word in the pair from one site was appended with suffix “m1,” and the word from the other site was appended with “_m2.”

For all scenarios, we used PDPS to predict test patients’ diagnoses in the final admission given all their records before the final admission. As an evaluation measure, we used the area under the receiver operating characteristic curve (AUC), for which 1 represents a perfect model, and 0.5 represents a worthless model. The average AUCs of a variety of diagnoses of different models were compared, and the benefit of harmonization is shown in the following three scenarios.

#### Incomplete Information

To evaluate the performance of the Procrustes harmonization, we first looked at how well missing medical events in one site can be compensated with the event vectors from another site. Oftentimes, small clinics or hospitals might not have encountered all medical events. In terms of PDPS prediction, missing events with no embeddings cannot be incorporated into the making of the patient vector, making prediction less accurate. Moreover, prediction simply cannot be made with PDPS for certain diagnoses if there are no embeddings for those diagnoses.

**Figure 5:**
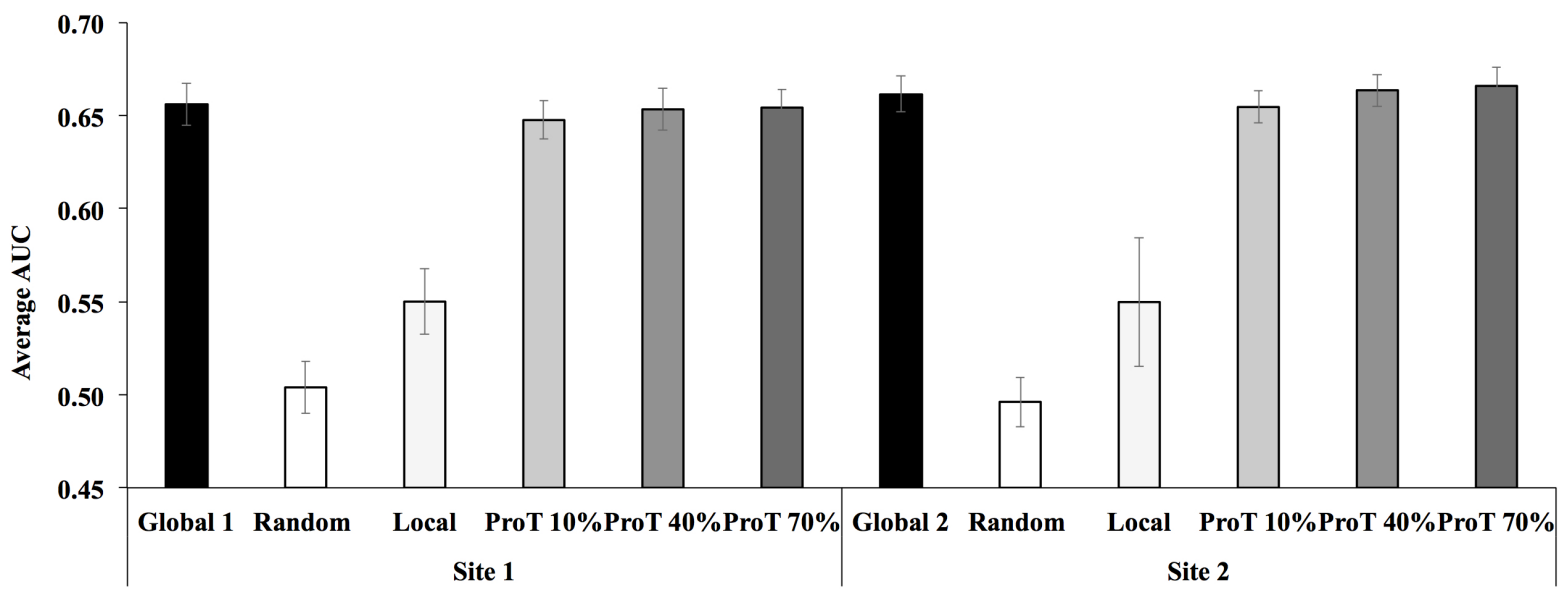
Average area under the receiver operating characteristic curve (AUC) of several different scenarios. Site1 had no vectors for a set of 20 diagnoses, whereas site 2 had no vectors for another set of 20 diagnoses. Using global model, we predicted on the 20 diagnoses missing from site 1 (Global 1) and the 20 diagnoses missing from site 2 (Global 2). The missing vectors were compensated with either random vectors (random), untransformed vectors from the other site (local), or Procrustes harmonized vectors from the other site that were harmonized using different percentage of corresponding pairs (ProT 10%, 40%, and 70%).

To resolve such a problem, we compensated the missing information using diagnosis vectors from another hospital. To test whether diagnosis vectors from another hospital can be used accurately to predict in another site, we first took the 40 most common diagnoses in MIMIC-III and randomly separated them into two sets of 20 diagnoses. The two “local” sites both originally had all 40 diagnoses, but we took one “local” site and deleted all instances of one set of 20 diagnoses (Multimedia Appendix 9, colored blue) and took the other “local” site and deleted the other set of 20 diagnoses (Multimedia Appendix 9, colored red). We trained and contextually embedded these two raw datasets separately, making two embedding models where each was missing a different set of 20 diagnoses. Figure 5, *Global 1*, shows that the average AUC of the 20 diagnoses that were missing from site 1 predicted using the global embedding model; Figure 5, *Global 2,* shows that the average AUC of the 20 diagnoses that were missing from site 2 predicted using the global model. These two act as the baselines. Then we explored what vectors added to the “local” embeddings could compensate for the missing diagnoses vectors. When we simply added random vectors for the 20 missing diagnoses to the respective sites and predicted using the “local” models, the average AUC for those missing diagnoses for both sites was close to 0.5 (Figure 5, *Random*). If we compensated vectors for the missing diagnoses with vectors in the other site without Procrustes harmonizing the embeddings of two sites first, the average AUC only improved to approximately 0.55 (Figure 5, *Local*). However with harmonization, compensating the missing diagnoses vectors with vectors from the other site returned the AUC to the level of the global model (Figure 5 Procrustes Transformed, *ProT*). We also tested whether using different percentage of corresponding anchor pairs for Procrustes would alter the AUC. Figure 5 shows that increasing the percentage of corresponding pairs increases the AUC in a very negligible manner.

#### Split Patient History

In another scenario, it is conceivable that patients may go to different hospitals, leaving parts of their patient history in one hospital while other parts in other hospitals. One can simply predict future events of these patients using part of their clinical pathway at each site, but a more accurate prediction can be made from his or her entire clinical pathway. However, obtaining the entire clinical pathway is not easy. First, it might be time-consuming or even infeasible to release patient history across hospital sites. Second, even if the entire patient history is in one site, the events in a patient’s clinical pathway may be coded differently from site to site, leading to some events being unrecognizable by a model built solely on one site and unusable for prediction. To solve these two problems, hospitals can first share their own contextual embeddings and combine them into a common space using Procrustes. Then, for all the patients who have history in multiple hospitals, each local clinical pathway can be made into a local patient vector, effectively rendering the history unidentifiable. Finally, every local patient vector can be summed and normalized to obtain an approximation of the global patient vector. Then prediction can be conducted using the approximated global patient vectors and diagnoses vectors in each “local” hospital. The following experiment shows that the initial harmonization step is required to obtain significant prediction results.

**Figure 6:**
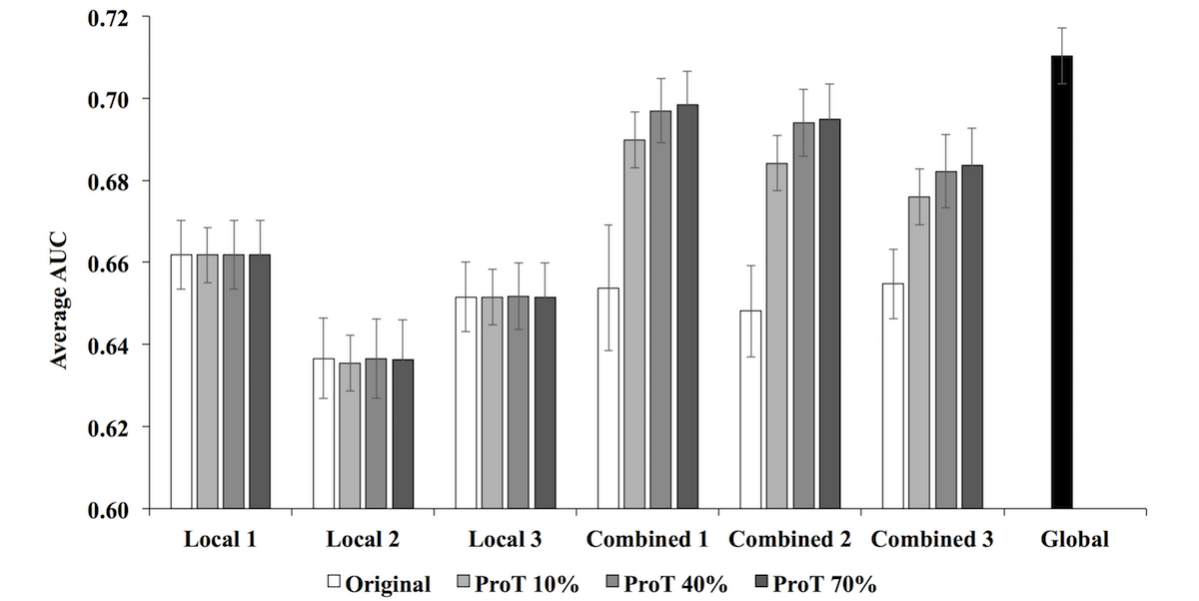
Average area under the receiver operating characteristic curve (AUC) of the 80 most common diagnoses. Local 1, 2, and 3 show local sites using local embedding models either unharmonized (Original) or harmonized (Procrustes Transformed, ProT) and using only the part of the clinical pathway of test patients in the respective hospital. Combined 1, 2, and 3 show all three local test patient vectors combined, where each local vector is made with locally learned event vectors either unharmonized (Original) or harmonized (ProT).

For this task, we divided the raw MIMIC-III training set into three “local” sites then trained three “local” embedding models. For medical events in each “local” site 1, 2, and 3, suffixes “_m1,” “_m2,” and “_m3” were added to the end, respectively, simulating that each “local” site used their own coding system. To simulate test patients who have records in three “local” sites, we divided the clinical pathway of each test patient into three sections, where each section was appended with suffix “_m1,” “_m2,” or “_m3” to designate which section belonged to which site. The average AUC of the 80 most common diagnoses from MIMIC-III (Multimedia Appendix 9) was evaluated based on PDPS for different models and is shown in Figure 6. The average AUCs calculated with “local” site 1, 2, and 3 embedding models (*Original of Local 1, 2, and 3*) dropped significantly compared with the global model (*Global*). This was because each “local” model could only use one-third of the information of each test patient for prediction. If we summed and normalized the three local test patient vectors together without harmonizing the embeddings first, the AUC did not improve (*Original of Combined 1, 2, and 3*). However, when the three contextual embeddings were harmonized with Procrustes (*ProT*) first, summing and normalizing the local test patient vector together improved the AUC closer to the AUC of prediction made by the global model (*ProT of Combined 1, 2, and 3*). Figure 6 shows three “combined” results because each local site had its own diagnosis vectors that PDPS and subsequent AUC were calculated based on. We tested different percentages of corresponding pairs of 10%, 40%, and 70% for Procrustes harmonization. Similar to the previous experiment, Figure 6 shows that increasing the percentage of corresponding pairs had positive but negligible effect.

#### Hospitals With Different Sizes

**Figure 7:**
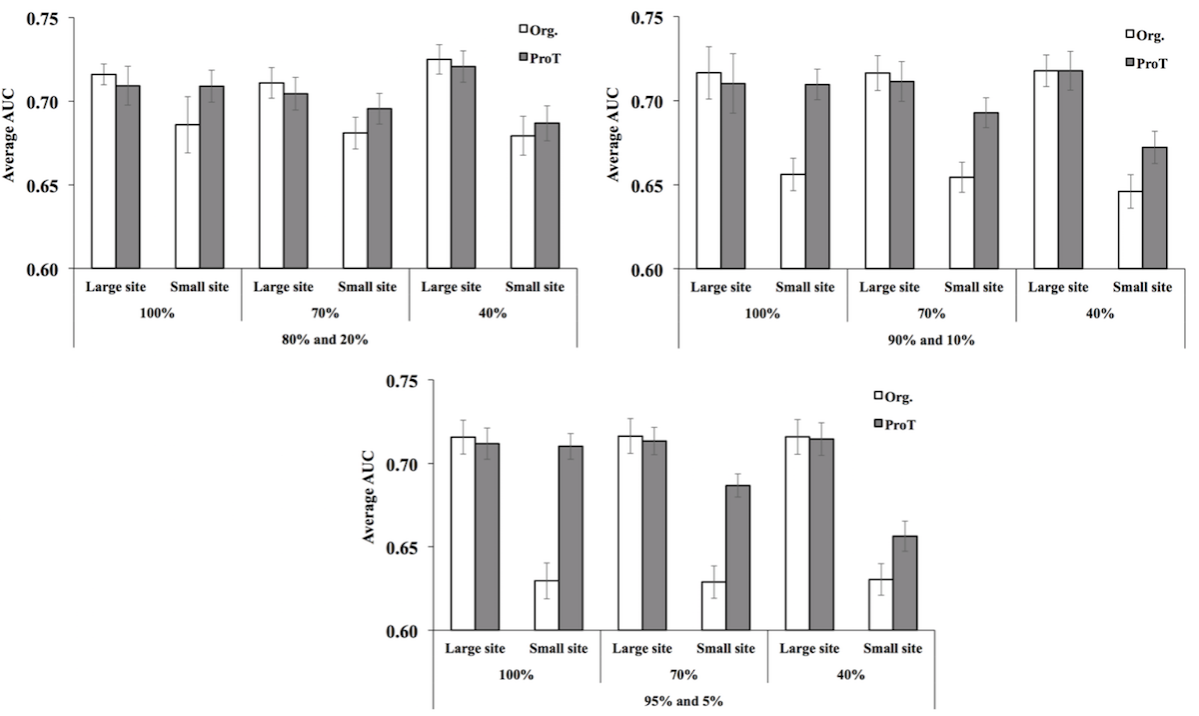
Average area under the receiver operating characteristic curve (AUC) of 80 most common diseases in Medical Information Mart for Intensive Care III (MIMIC-III) for hospital of different sizes harmonized using different percentage of corresponding pairs: upper left: 80% versus 20%, upper right: 90% versus 10%, bottom: 95% versus 5%.

Another possible scenario is that hospitals have different sizes. One hospital might be much smaller than the other. The smaller hospital might not be able to predict diseases of new patients accurately based on its existing patient history because small hospitals have limited or skewed data. In this case, our alignment can help the small hospital overcome such a limitation by incorporating information from the larger hospital. To test this scenario, we split MIMIC-III raw data into two sites with imbalanced ratios of hospital sizes that varied from 80% and 20%, 90% and 10%, to 95% and 5%. We also used different ratios of corresponding anchor pairs from 40%, 70%, to 100% of all possible corresponding pairs. After harmonization, we introduced one more simple task called fusion to boost the prediction performance in the small hospital. If an event was included in the anchor pairs, we took the weighted average depending on the size of the hospital to combine the two event vectors into one vector. If the event was not included, we found the nearest neighborhood (ie, *k*=1 where *k* is the number of nearest neighborhoods) from the other site and averaged itself and the nearest neighborhood. After harmonization and fusion, the average AUC of the most common 80 diagnoses for each combination was calculated. Figure 7 shows AUC results of large and small hospitals with or without harmonization and different ratios of corresponding pairs. A larger difference in hospital size results in a larger difference in AUC, meaning the more information there is, the more accurate the model is. Furthermore, Figure 7 shows that a small hospital can improve its prediction performance without compromising the integrity of the larger hospitals through harmonization and fusion with data from large hospitals, which is an amenable feature.

### Unstructured Data Results

Our harmonization method can be extended to unstructured clinical notes. Using the same method as structured data, we built a clinical pathway for unstructured data, except we used medical concepts extracted from Metamap [27]. Then we built a global embedding model and two “local” embedding models. Finally, we harmonized the two “local” model with Procrustes. There were approximately 150,000 unique medical concepts extracted with Metamap, of which approximately 72,000 were common in both sites that could be used as anchor pairs. For anchor pairs, we used the top 10% most common occurring corresponding pairs. These were common concepts such as “admission,” “alter,” “recalls,” etc. The most common occurring anchor pairs were chosen because they were the most likely to have a similar neighborhood and structure relative to other concepts in each “local” hyperdimensional space, giving us the most reliable transformation matrix *Q* for equation 1. For some of the experiments, we had to create patient vectors. We used the same method as the method for structured data, but we omitted the time decay factor (*λ*). The following experiments were conducted to demonstrate the benefit of harmonized local embedding models.

#### Concept Unique Identifier Group Distances

**Figure 8:**
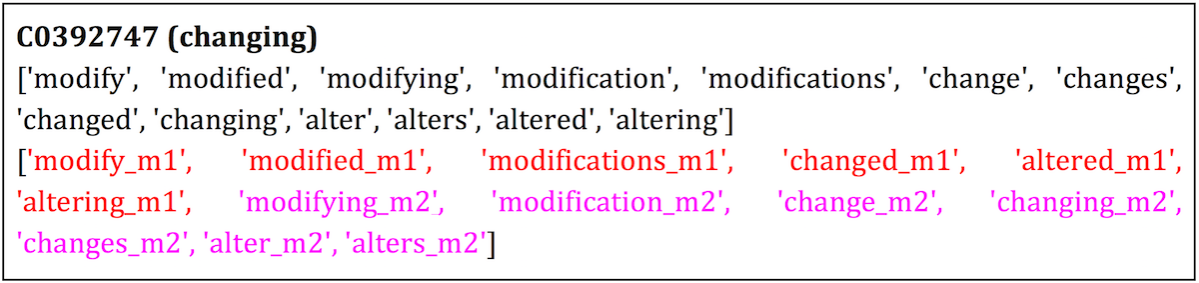
(a) Example of medical concepts belonging to a concept unique identifier (CUI) groups from a global model. (b) Red words were found from site 1 “local” model, magenta words were found from site 2 “local” model.

For unstructured data, the clinical pathways used to train Word2Vec model consist of Metamap concepts [27]. Each for these concepts belongs to a concept unique identifier (CUI) created by the Unified Medical Language System, and each CUI may contain many concepts. For example, in the top image of Figure 8, there are many words that belong to the CUI C0392747, which is about “changing.” We calculated the average pairwise cosine similarity among all members within a CUI group using concept vectors from the global model and found the average similarity for all CUI groups to be approximately 0.42. For the local models, within each CUI group, some words were learned from site1 local model (designated with the suffix “_m1”), whereas some words were learned from site 2 local model (designated with the suffix “_m2”). For common words that appeared in both sites, we randomly assigned them with either vectors from site 1 “local” model or vectors from site 2 “local” model. Again, we calculated the average pairwise cosine similarity among all members within each CUI group. If site 1 and site 2 models were not harmonized with Procrustes, the average similarity dropped to approximately 0.23, but the average similarity returned to approximately 0.42 if the local models were harmonized. Furthermore, if we limit the words in both sites to only include top 10% of the most common words, the average similarity after harmonization further increase to approximately 0.62, whereas the similarity without harmonization remains approximately 0.24. In addition, experiment was repeated with two real datasets. We used the MIMIC-III dataset to build site 1 “local” model and i2b2 dataset [32-36] to build site 2 “local” model. The results showed similar pairwise cosine similarity as the artificial datasets results.

This demonstrates that words that are part of the same CUI group but learned from separate local sites can be combined and have distances restored to the global level with harmonization at a concept embedding level. Next, we will explore harmonization at the patient level.

#### Patient Similarity

For the following experiments, we attempted to explore patient similarity. We created patient vectors with the “global” embedding model and found pairs of patients who were the most similar in terms of the cosine similarity. We then separated these patients into two local sites and trained embedding models separately to evaluate most similar patients’ retrieval from the other site. After making patient vectors for the two “local” sites, we took all patients from one site and calculated the rank of their most similar patients previously found using the global model, which was then in the other “local” site. Without Procrustes harmonizing the embedding models, the average rank of the most similar patient previously found in the global model dropped to approximately 1035.89 in the other site. However, with harmonization, the average rank for the most similar patient previously found rose back to approximately 1.35 in the other site. This demonstrates that it’s difficult to find the most similar patient across site without harmonizing the embeddings of the sites first.

Even though we were able to restore the most similar patients that were found in the global model, we still needed to demonstrate that the most similar patients found after harmonization is relevant in terms of prediction. Therefore, we designed the following experiment to see if missing information can be compensated with information from other sites that is harmonized. To predict for future diagnosis in this experiment, PDPS was not used because it required diagnoses vectors, which, for the structured data, were ICD-9 diagnosis codes learned during contextual embedding. However, contextual embedding for unstructured data was done on clinical notes that did not contain any ICD-9 codes.

**Figure 9:**
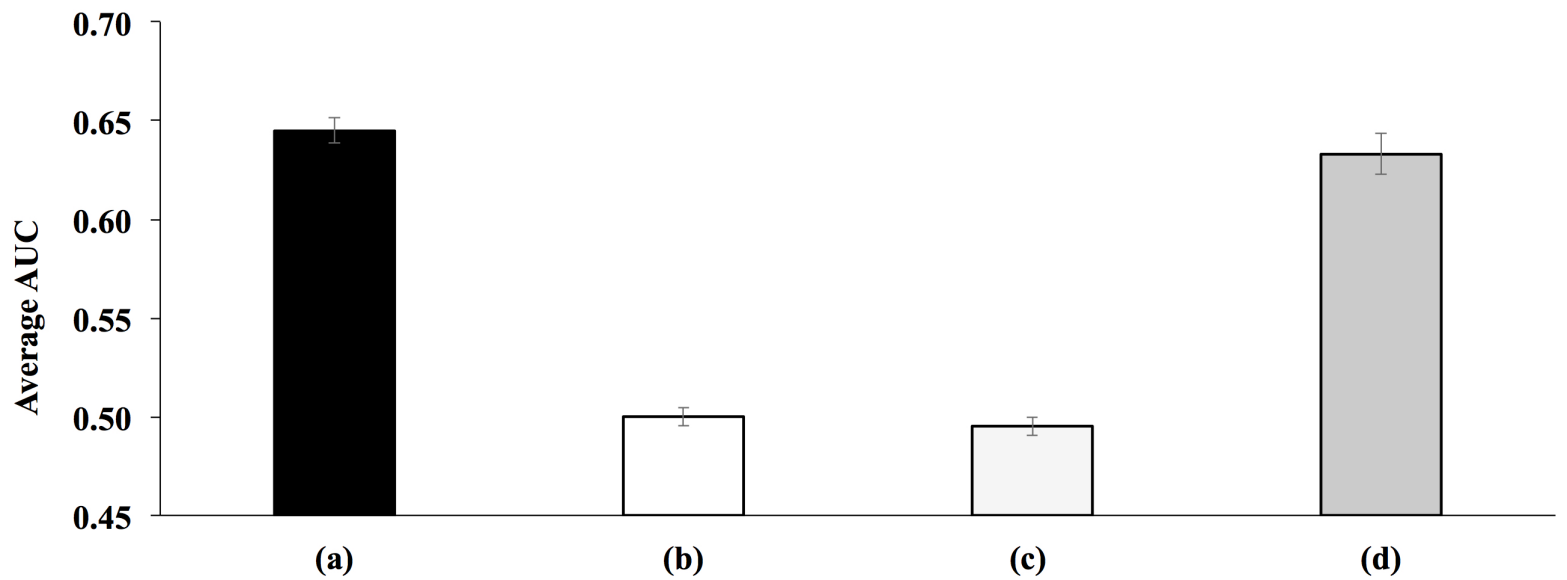
Average area under the receiver operating characteristic curve (AUC) over 80 most common diagnoses with each taking turn acting as the diagnosis of interest. Site 1 contained patients with diagnosis of interest, whereas site 2 did not. (a) Patient vectors of new test patients were created with embeddings 1, and training patients vectors of site 1 were used to find most similar patients for new test patients and to predict the diagnosis of interest. (b) Patient vectors of new test patients were created with embeddings 2, and training patients vectors of site 2 were used. (c) Patient vectors of new test patients were created with embeddings 2, and training patients vectors of site 1 were used. (d) Embeddings of site 1 and site 2 were harmonized before creating patient vectors. Then, patient vectors of new test patients were created with embeddings 2, and training patients vectors of site 1 were used to find most similar patients for new test patients.

Instead, we used the most similar training patients to each test patient to predict test a patient’s future diagnosis. To find the most similar patients for each test patient, we calculated the average cosine similarity between a test patient to the training patients. Then, we used the training patients whose cosine similarities were one SD above the average as the most similar patients. Finally, prediction and subsequent AUC were calculated by probabilities from voting using the most similar training patients and their true ICD-9 diagnoses from the structured data as labels.

After learning contextual embeddings for the two “local” sites, we again created training patient vectors for the two sites. Then, in site 2, we deleted all patients with a certain diagnosis of interest but retained patients with this diagnosis in site 1. This experiment was done for the 80 most common diagnoses in MIMIC-III, with each taking turn acting as the diagnosis of interest. The average AUC over these 80 diagnoses is shown in Figure 9. Given a set of test patients, their patient vectors were created using either embeddings from site 1 or embeddings from site 2 depending on which hospital they were admitted to. The first column of Figure 9 shows the result where new test patients were admitted to site 1, and their patient vectors were created with site 1 embeddings. We predicted whether these new patients would develop the diagnosis of interest based on the most similar training patients in site 1 and obtained reasonable results. However, when new test patients were admitted to site 2, and we used the most similar training patients in site 2 to predict the probability of developing the diagnosis of interest, the result was no better than guessing as shown in the second column of Figure 9, because site 2 did not contain patients with the diagnosis of interest. Similarly, when new test patients were admitted to site 2, and we used the most similar patients found from site 1 to form a prediction, the result was no better than guessing as shown in the third column of Figure 9 because the embedding space was not harmonized, and not enough relevant similar patients were found. Finally, we harmonized the “local” embeddings between site 1 and site 2 first and created training patients vectors from them. When new test patients were admitted to site 2 and created their patient vectors with the harmonized “local” embedding of site 2, we could then use site 1 to find reasonable most similar patients and obtain significant AUC as shown in the fourth column of Figure 9. This shows that if new patients were admitted to a hospital and found a lack of relevant most similar patients to reasonably make accurate predictions on a diagnosis of interest, patient record from another hospital could compensate and provide relevant most similar patients. However, such compensation could only be achieved if contextual embeddings were harmonized between the hospitals.

## Discussion

### Principal Findings

This paper serves as a proof-of-concept that contextual embedding models, which are becoming bedrocks to deep learning analysis in place of one-hot representations, can be harmonized and subsequently synchronize information from different hospital sites for better prediction capability without sacrificing privacy. However, one limitation of our work is that all experiments were conducted on a single MIMIC-III database. The underlying structure of the simulated local models may be similar, making it easy to approximate the global model from combining harmonized models. However, we argue that every hospital will have similar structures and relationships for medical events or concepts related to diagnoses that are common and widespread. Using events related to these common diagnoses, we can nevertheless derive a reasonable transformation matrix to apply to the rest of the data even if we extend the method beyond the MIMIC-III database. Ultimately, harmonization can bring knowledge specialized in each hospital into the same space. This is a major benefit because once embeddings are created for each medical event or concept, it is difficult to add new event or concepts in relation to the existing embeddings without training the model again. With harmonization, we can leverage embeddings learned from another source and add new vectors. At the moment, what this process fails to address are instances when two hospitals have conflicting embeddings regarding an event or concept. This method does not alleviate the issue but simply leaves both embeddings. The fusion method mentioned in the experiment conducted on hospitals with different sizes somewhat explored the issue. However, to truly create a global model where these two embeddings harmonize into one, further work is required. The current method works best to incorporate new information that hospitals are missing, whether it is missing diagnoses or parts of patient’s clinical pathway.

The portability of event and patient vectors is another major strength of the harmonization method. With event and patient vectors rendered to vectors of numbers, privacy is preserved, yet information is still conveyed to hospitals involved in the harmonization. Instead of preserving privacy through deidentification and encryption, we take more of a machine-learning approach, where we tackle privacy protection and the sharing of data simultaneously. Currently, the way patient vectors are created is relatively naive, especially for unstructured data. However, the explosion of deep neural networks, such as recurrent and convolutional neural networks, can create more sophisticated patient vectors. What we have shown is the need of harmonization to analyze patients vectors learned at different sites together. It would be interesting to see if harmonization can be applied to deep learning in a distributed manner.

Finally, we have shown in a limited way the extension of the harmonization method onto three sites, but we also see that even with harmonization, the prediction results do not reach the global level. Future work can explore the possibility of harmonizing more sites, where the number of corresponding pairs diminishes as the number of sites increases.

### Conclusions

Contextual embedding models are extremely useful in health care modeling because of their representativeness and applicability to downstream machine-learning models. With patient privacy being a paramount concern, it is nontrivial to directly share medical records in both structured and unstructured form. The emergence of contextual embedding in health care allows for a new way to share models without sharing data. We proposed an innovative framework to combine locally trained embeddings into embeddings in a global sense. Utilizing our unique harmonization, more accurate analyses can be made with the accumulated knowledge acquired from local sources. Such a technique can allow for information unique to a certain hospital to become available to other sites, increasing the fluidity of information flow in health care. Our demonstration is on Word2Vec, but it is widely applicable to other contextual embedding models, including the most recent Med2Vec [20] and Graph2Vec [37].

## Appendix

Multimedia Appendix 1 CBOW replicate for Figure 5.

Multimedia Appendix 2 GloVe replicate for Figure 5.

Multimedia Appendix 3 CBOW replicate for Figure 6.

Multimedia Appendix 4 GloVe replicate for Figure 6.

Multimedia Appendix 5 CBOW replicate for Figure 7.

Multimedia Appendix 6 GloVe replicate for Figure 7.

Multimedia Appendix 7 CBOW replicate for Figure 9.

Multimedia Appendix 8 GloVe replicate for Figure 9.

Multimedia Appendix 9 List of 80 most common diagnoses used for prediction. Diagnoses that are colored blue are diagnoses that were deleted from Site 1 in Section 3.2.1, while diagnoses that are colored red are diagnoses that were deleted from Site 2.

## Abbreviations

AUC: area under the receiver operating characteristic curve
CBOW: continuous bag-of-words
CUI: concept unique identifier
EHR: electronic health record
HIPAA: Health Insurance Portability and Accountability Act
ICD: International Classification of Diseases
MIMIC-III: Medical Information Mart for Intensive Care-III
ProT: Procrustes Transformed
