# Supplementary figures and images for: Privacy-Preserving Predictive Modeling: Harmonization of Contextual Embeddings From Different Sources
Source: JMIR Med Inform. 2018 May 16;6(2):e33. doi: 10.2196/medinform.9455 (PMC5981054; doi:10.2196/medinform.9455)

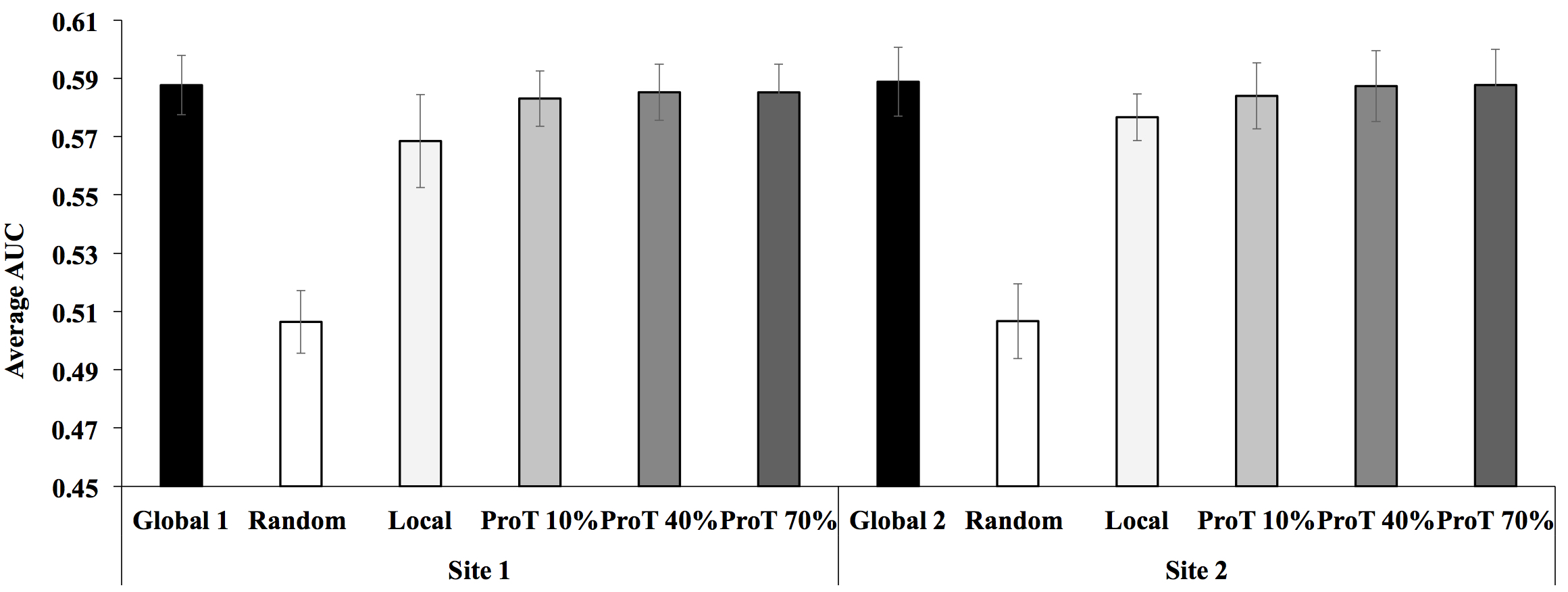

Supplement: Multimedia Appendix 1 [file medinform_v6i2e33_app1.jpg]

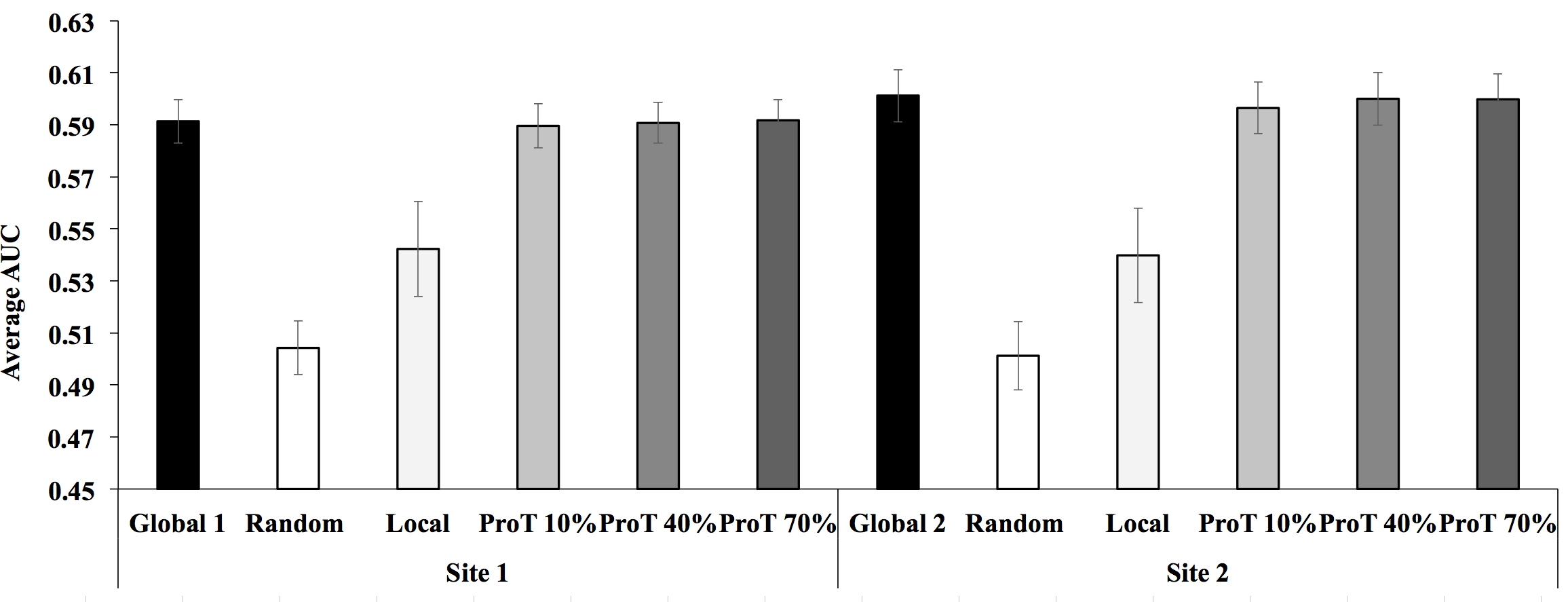

Supplement: Multimedia Appendix 2 [file medinform_v6i2e33_app2.jpg]

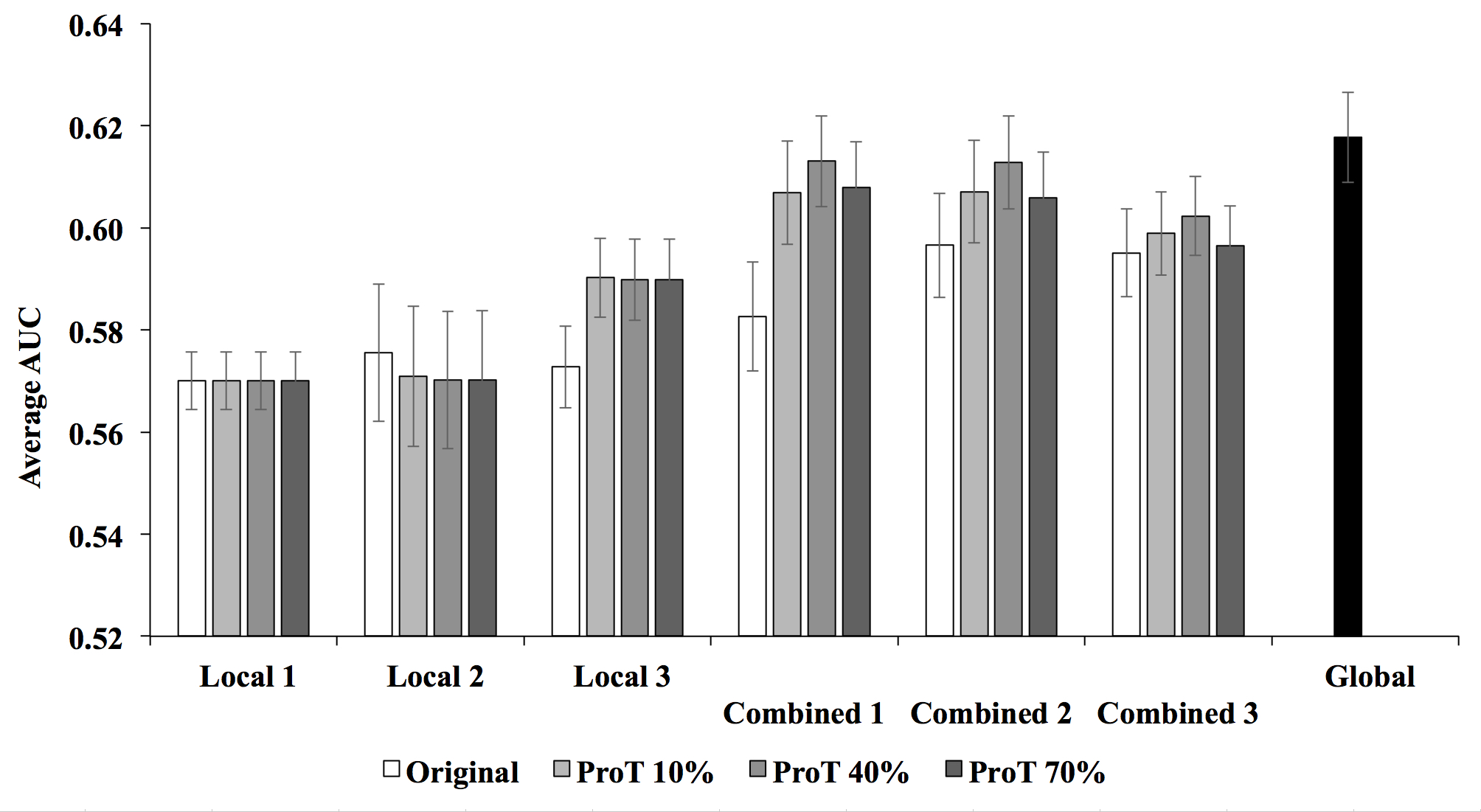

Supplement: Multimedia Appendix 3 [file medinform_v6i2e33_app3.jpg]

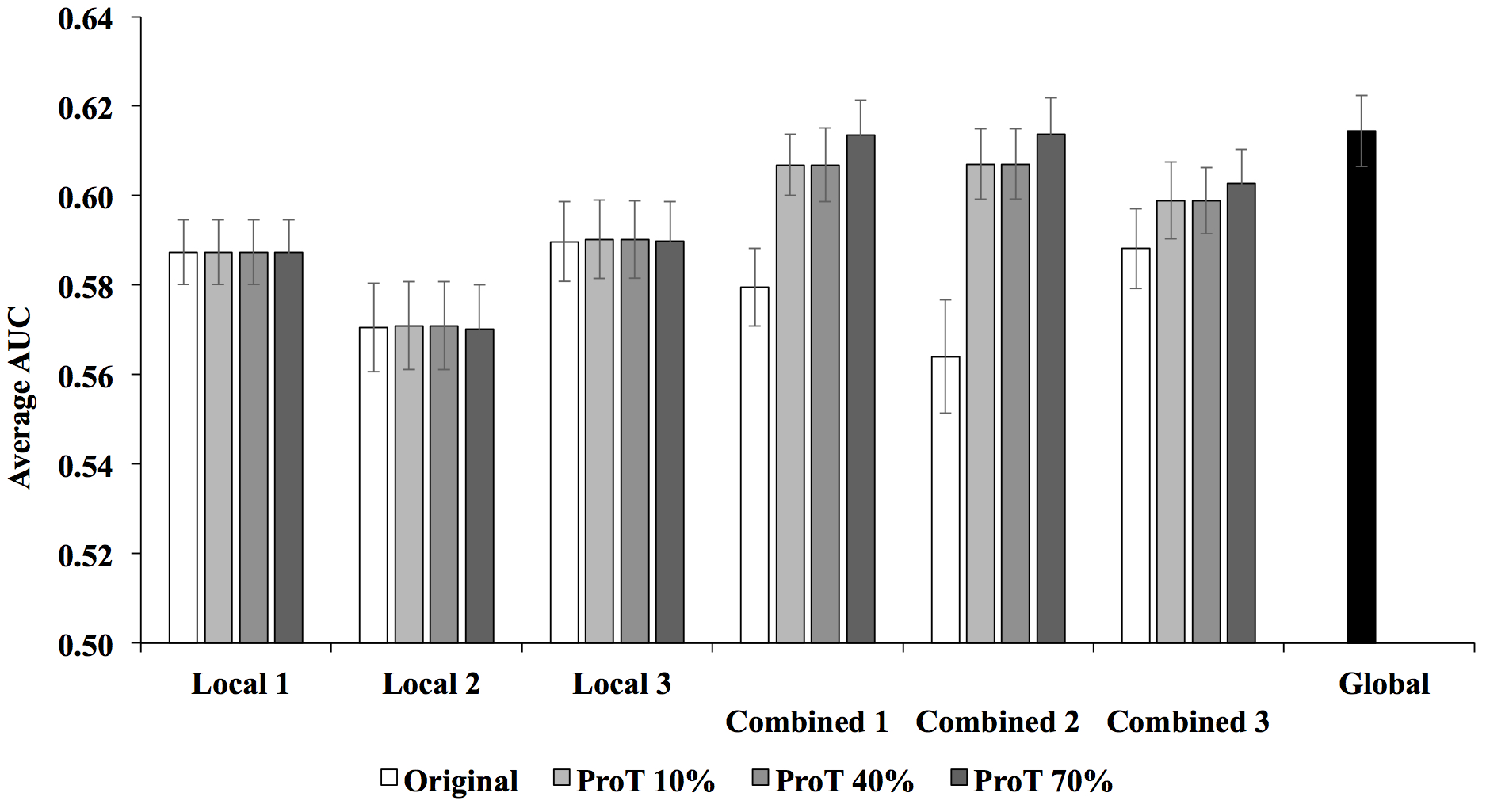

Supplement: Multimedia Appendix 4 [file medinform_v6i2e33_app4.jpg]

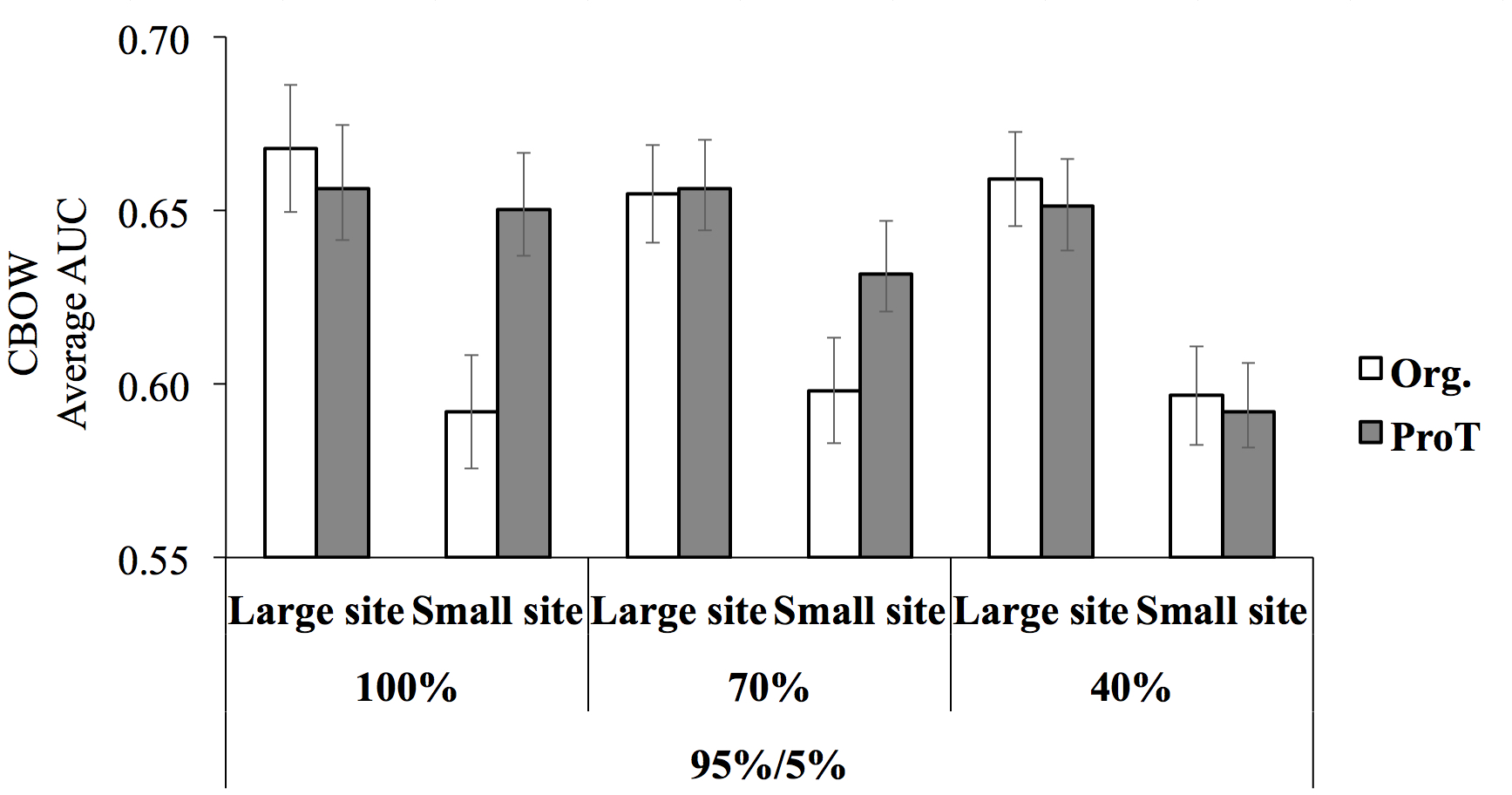

Supplement: Multimedia Appendix 5 [file medinform_v6i2e33_app5.jpg]

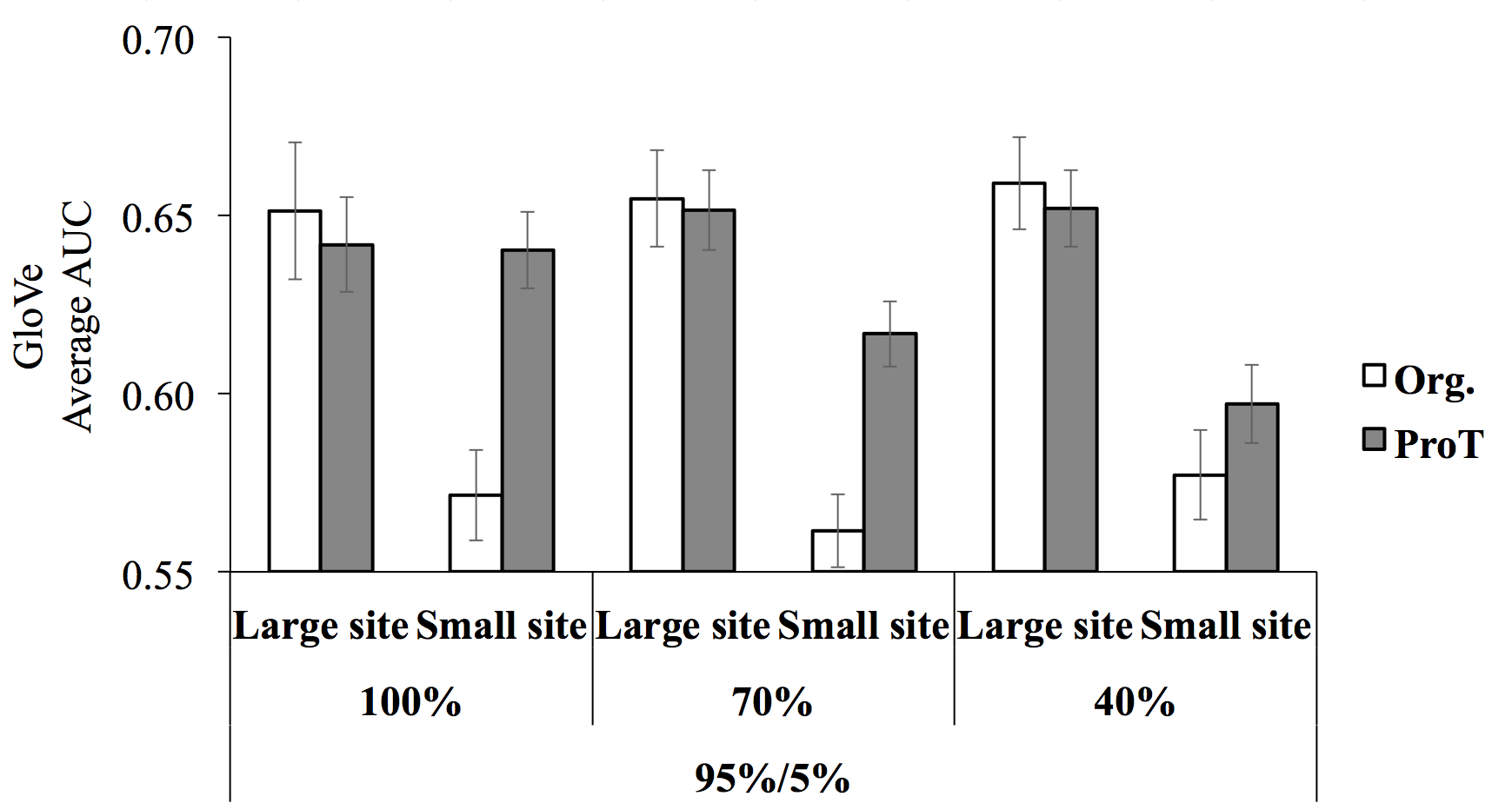

Supplement: Multimedia Appendix 6 [file medinform_v6i2e33_app6.jpg]

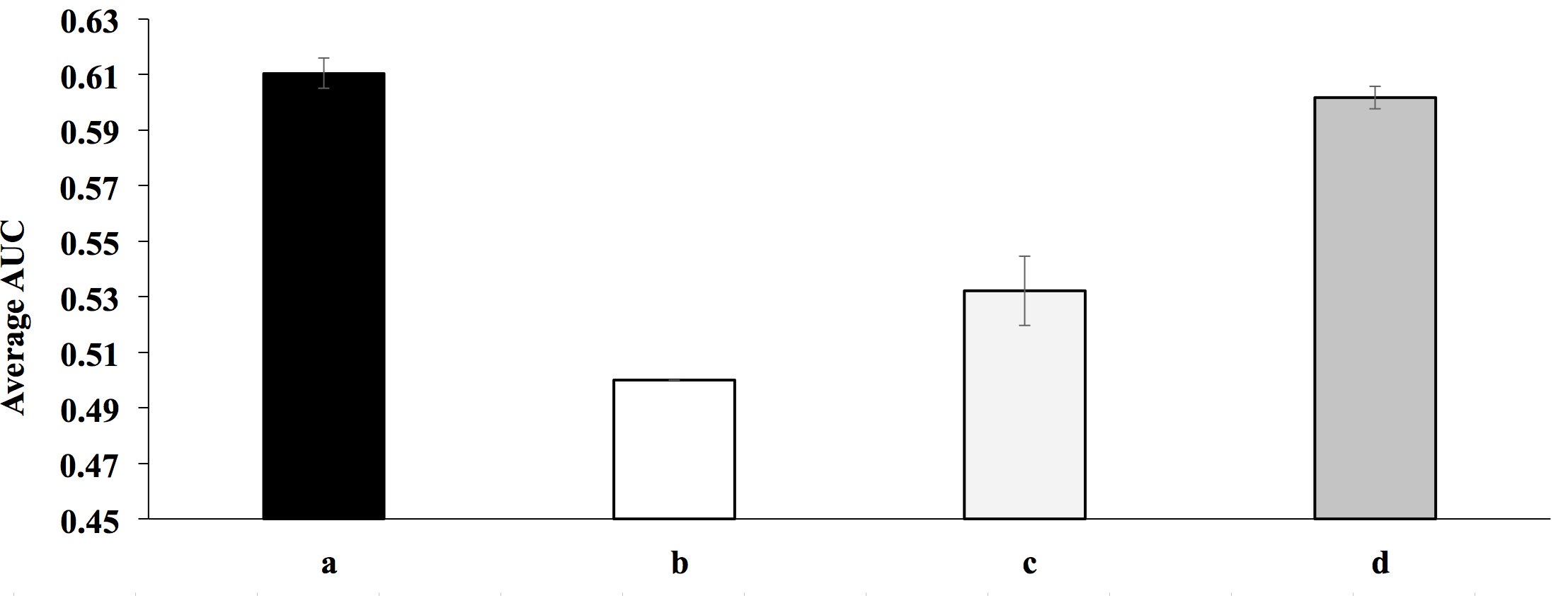

Supplement: Multimedia Appendix 7 [file medinform_v6i2e33_app7.jpg]

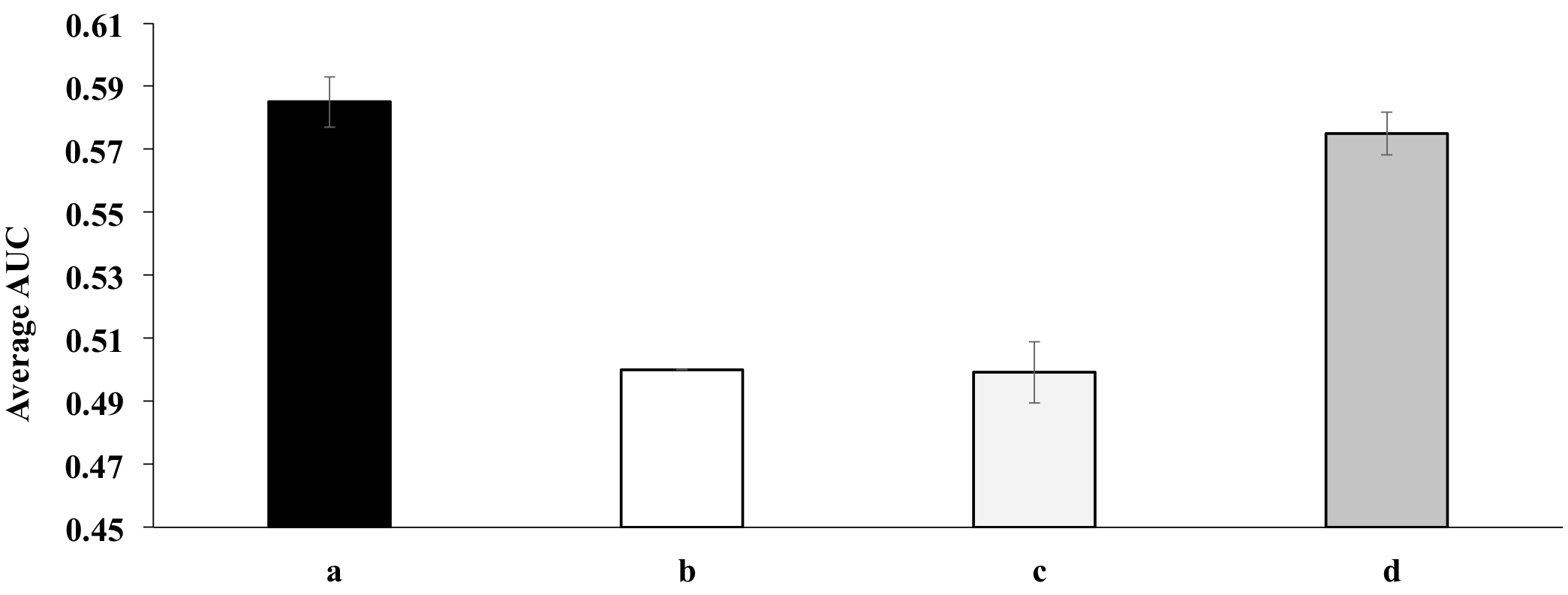

Supplement: Multimedia Appendix 8 [file medinform_v6i2e33_app8.jpg]
