# Supplementary material for: Privacy-Preserving Predictive Modeling: Harmonization of Contextual Embeddings From Different Sources
Source: JMIR Med Inform. 2018 May 16;6(2):e33. doi: 10.2196/medinform.9455 (PMC5981054; doi:10.2196/medinform.9455)
Supplement: Multimedia Appendix 9 [file medinform_v6i2e33_app9.pdf]

Appendix A. List of 80 most common diagnoses used for prediction. Diagnoses that are colored blue are diagnoses that were deleted from Site 1 in **Section 3.2.1**, while diagnoses that are colored red are diagnoses that were deleted from Site 2.

**ICD**	**Description**
008	Intestinal infections due to other organisms
038	Septicemia
041	Bacterial infection in conditions classified elsewhere and of unspecified site
070	Viral hepatitis
112	Candidiasis
197	Secondary malignant neoplasm of respiratory and digestive systems
198	Secondary malignant neoplasm of other specified sites
244	Acquired hypothyroidism
250	Diabetes mellitus
263	Other and unspecified protein-calorie malnutrition
272	Disorders of lipoid metabolism
274	Gout
275	Disorders of mineral metabolism
276	Disorders of fluid, electrolyte, and acid-base balance
278	Overweight, obesity and other hyperalimentation
280	Iron deficiency anemias
285	Other and unspecified anemias
286	Coagulation defects
287	Purpura and other hemorrhagic conditions
288	Diseases of white blood cells
293	Transient mental disorders due to conditions classified elsewhere

294	Persistent mental disorders due to conditions classified elsewhere
300	Anxiety, dissociative and somatoform disorders
303	Alcohol dependence syndrome
305	Nondependent abuse of drugs
311	Depressive disorder, not elsewhere classified
327	ORGANIC SLEEP DISORDERS
338	PAIN
345	Epilepsy and recurrent seizures
348	Other conditions of brain
357	Inflammatory and toxic neuropathy
362	Other retinal disorders
401	Essential hypertension
403	Hypertensive chronic kidney disease
410	Acute myocardial infarction
412	Old myocardial infarction
414	Other forms of chronic ischemic heart disease
416	Chronic pulmonary heart disease
424	Other diseases of endocardium
425	Cardiomyopathy
427	Cardiac dysrhythmias
428	Heart failure
438	Late effects of cerebrovascular disease
440	Atherosclerosis
441	Aortic aneurysm and dissection
443	Other peripheral vascular disease

453	Other venous embolism and thrombosis
456	Varicose veins of other sites
458	Hypotension
482	Other bacterial pneumonia
486	Pneumonia, organism unspecified
491	Chronic bronchitis
493	Asthma
496	Chronic airway obstruction, not elsewhere classified
507	Pneumonitis due to solids and liquids
511	Pleurisy
518	Other diseases of lung
519	Other diseases of respiratory system
530	Diseases of esophagus
560	Intestinal obstruction without mention of hernia
562	Diverticula of intestine
564	Functional digestive disorders, not elsewhere classified
569	Other disorders of intestine
571	Chronic liver disease and cirrhosis
572	Liver abscess and sequelae of chronic liver disease
577	Diseases of pancreas
578	Gastrointestinal hemorrhage
584	Acute renal failure
585	Chronic kidney disease (CKD)
599	Other disorders of urethra and urinary tract
600	Hyperplasia of prostate

682	Other cellulitis and abscess
707	Chronic ulcer of skin
715	Osteoarthrosis and allied disorders
733	Other disorders of bone and cartilage
995	Certain adverse effects not elsewhere classified
996	Complications peculiar to certain specified procedures
997	Complications affecting specified body systems, not elsewhere classified
998	Other complications of procedures, NEC
E8798	Abn react-procedure NEC
